# Neutrophil heterogeneity and aging: implications for COVID-19 and wound healing

**DOI:** 10.3389/fimmu.2023.1201651

**Published:** 2023-11-28

**Authors:** Yi Liu, Changlan Xiang, Zhenni Que, Chenglong Li, Wen Wang, Lijuan Yin, Chenyu Chu, Yin Zhou

**Affiliations:** Department of Hematology, Sichuan Provincial People's Hospital, University of Electronic Science and Technology of China; Medical Cosmetic Center, Chengdu Second People's Hospital; Minhang Hospital, Fudan University, Shanghai, China

**Keywords:** neutrophil, aging, heterogeneity, COVID-19, wound healing, immune response

## Abstract

Neutrophils play a critical role in the immune response to infection and tissue injury. However, recent studies have shown that neutrophils are a heterogeneous population with distinct subtypes that differ in their functional properties. Moreover, aging can alter neutrophil function and exacerbate immune dysregulation. In this review, we discuss the concept of neutrophil heterogeneity and how it may be affected by aging. We then examine the implications of neutrophil heterogeneity and aging for COVID-19 pathogenesis and wound healing. Specifically, we summarize the evidence for neutrophil involvement in COVID-19 and the potential mechanisms underlying neutrophil recruitment and activation in this disease. We also review the literature on the role of neutrophils in the wound healing process and how aging and neutrophil heterogeneity may impact wound healing outcomes. Finally, we discuss the potential for neutrophil-targeted therapies to improve clinical outcomes in COVID-19 and wound healing.

## 1 Introduction

Neutrophils are critical immune cells that play a vital role in the body's response to infection and tissue injury (1). However, recent studies have identified that these cells are a heterogeneous population with distinct subtypes that exhibit unique functional properties (2, 3). Recent scRNA-seq research reveals significant heterogeneity among neutrophils, contradicting prior views of their homogeneity. By analyzing thousands of mouse neutrophils, eight distinct subsets were identified, each with unique functions and maturation processes under normal and infectious conditions. Bacterial infection primes these subsets for enhanced activity without disrupting their heterogeneity, facilitating deeper exploration of neutrophil-related diseases, biomarkers, and therapies at a single-cell resolution (4, 5). Recent research has revealed neutrophil heterogeneity, with CD66b(+) cells exhibiting neutrophil-like morphology in inflammation. These cells possess immunosuppressive or proinflammatory properties and are referred to as LDNs, LDGs, G-MDSCs, or immunosuppressive neutrophils. However, due to the absence of specific markers, their precise phenotype and function remain unclear. This article provides an overview of mature and immature neutrophil subsets with immunosuppressive or proinflammatory characteristics, addressing unresolved questions and gaps in our understanding of neutrophil heterogeneity (6, 7). Aging is known to have an impact on neutrophil function, leading to immune dysregulation, which may contribute to the severity of certain diseases (8). In this review, we aim to discuss the concept of neutrophil heterogeneity and its relationship with aging, specifically examining how it may impact COVID-19 pathogenesis and wound healing (9). We will provide an overview of the current understanding of neutrophil involvement in COVID-19, including the potential mechanisms underlying neutrophil activation and recruitment (10). Additionally, we will summarize the role of neutrophils in the wound healing process and discuss how aging and neutrophil heterogeneity may influence wound healing outcomes (11). Finally, we will review the potential of neutrophil-targeted therapies to improve clinical outcomes in COVID-19 and wound healing (12). Overall, a better understanding of neutrophil heterogeneity and its impact on immune function and disease pathogenesis may lead to the development of more targeted and effective therapies (13). Neutrophil heterogeneity is a relatively new concept that has emerged in recent years, and there is still much to be learned about the functional diversity of these cells (14). However, the potential clinical implications of this heterogeneity are significant (15). For example, recent studies have suggested that certain subtypes of neutrophils may be more effective at clearing infections, while others may contribute to tissue damage and inflammation (16). This raises the possibility of selectively targeting specific neutrophil subtypes for therapeutic purposes, which may be particularly relevant in the context of COVID-19, where an uncontrolled immune response can contribute to disease severity (17). Moreover, the dysregulation of neutrophil heterogeneity may contribute to the impaired wound healing seen in conditions such as diabetes, highlighting the need for novel approaches to modulate neutrophil function and heterogeneity (18). Overall, a better understanding of the complex biology of neutrophils and their heterogeneity will be crucial in developing new therapeutic strategies to improve clinical outcomes in a range of disease settings.

## 2 Neutrophil basics

Neutrophils are a type of white blood cell that play a crucial role in the immune system's response to infection and inflammation (19). These cells are the first responders to an infection, as they are rapidly recruited to the site of infection or injury in large numbers (20). Once they arrive at the site of infection, neutrophils use a variety of mechanisms to eliminate pathogens, including phagocytosis, the release of antimicrobial agents, and the formation of neutrophil extracellular traps (NETs) (21). Neutrophils also play a critical role in modulating the inflammatory response, as they release cytokines and chemokines that recruit other immune cells to the site of infection (22). However, in some cases, excessive neutrophil activation can contribute to tissue damage and exacerbate inflammatory diseases. Thus, understanding the basic biology of neutrophils is essential for developing new therapies for infections and inflammatory disorders (23). **Table 1** provides a comparison of the characteristics and functional properties of different neutrophil subtypes, highlighting the heterogeneity of these immune cells. In recent years, emerging research has shed light on the previously underappreciated ability of neutrophils to influence adaptive immunity (39). Neutrophils can interact with various immune cells, including dendritic cells, T cells, and B cells, and modulate their functions (40). This interaction suggests that neutrophils may play a more intricate role in shaping and regulating the adaptive immune response than previously believed (41). Recent studies have highlighted the capacity of neutrophils to induce the maturation of antigen-presenting cells (APCs) (42), particularly dendritic cells (DCs). Neutrophils can directly interact with DCs and promote their maturation by releasing pro-inflammatory cytokines, such as TNF-α and IL-12 (43). This activation of DCs leads to enhanced antigen presentation and subsequent activation of T cells, bridging the innate and adaptive immune responses (44). Notably, research has demonstrated that neutrophils can promote DC maturation in various infectious and inflammatory contexts (3, 45), providing valuable insights into the dynamic interplay between these immune cell populations. Recent progress in immunology has revealed an intriguing concept: neutrophils, traditionally considered as short-lived phagocytes, can also function as antigen-presenting cells (APCs). Neutrophils possess the ability to phagocytose pathogens, process the captured antigens (46), and subsequently present them on major histocompatibility complex class II (MHC-II) molecules, similar to classical APCs such as dendritic cells (42). This process involves the internalization of pathogens into neutrophil phagosomes, where the antigens are degraded and loaded onto MHC-II molecules. Consequently, neutrophils can engage with T cells, initiating adaptive immune responses and blurring the conventional boundaries between innate and adaptive immunity. These findings shed light on the versatile roles of neutrophils in orchestrating immune responses beyond their traditional phagocytic functions. The discovery that neutrophils can induce APC maturation and function as APCs themselves has significant implications for our understanding of immune responses and disease processes. This newfound role places neutrophils at the intersection of innate and adaptive immunity, highlighting their ability to bridge these two arms of the immune system. By directly influencing the maturation of APCs, neutrophils contribute to the initiation and regulation of adaptive immune responses. This finding opens up new avenues for exploring the dynamic interplay between neutrophils, APCs, and other immune cells, potentially leading to innovative therapeutic strategies for infectious diseases, autoimmunity, and cancer. Current research in the field of neutrophils functioning as APCs has provided valuable insights into the dynamic and complex nature of immune responses. However, there is still much to be explored and understood regarding the specific mechanisms and functional consequences of neutrophil-mediated APC maturation. Future studies could focus on elucidating the molecular pathways involved in this process, identifying the signals that trigger neutrophil transition into APCs, and investigating the impact of neutrophil-mediated APC maturation on various disease contexts. Continued research in this field holds immense potential to broaden our understanding of immune regulation and may pave the way for novel therapeutic interventions targeting immune-related disorders.

**Table 1:** comparing the characteristics of different neutrophil subtypes.

Neutrophil Subtype	Characteristics	Functional Properties	Reference
Low-density neutrophils (LDNs)	Lower density than classical neutrophils, segmented nuclei or bilobed nuclei	Increased cytokine production, pro-inflammatory phenotype, decreased phagocytic activity	(24, 25)
High-density neutrophils (HDNs)	Higher density than classical neutrophils, segmented nuclei	Increased phagocytic activity, higher oxidative burst capacity	(26–28)
Anergic neutrophils	Reduced cytokine production, increased apoptosis	Reduced ability to respond to stimuli	(29–31)
Hypersegmented neutrophils	Increased number of lobes in nucleus	Associated with vitamin B12/folate deficiency	(32–35)
Activated neutrophils	Increased CD11b and CD62L expression, increased ROS production	Increased ability to kill bacteria and fungi	(36–38)

### 2.1 Explain the basic characteristics of neutrophils and their life cycle

Neutrophils, a vital constituent of granulocytes or white blood cells, are indispensable to the innate immune response against infection and inflammation (47). They are recognized by their multilobed nuclei and cytoplasmic granules (48), that house an array of enzymes and antimicrobial agents (49). Known for their brief lifespan, ranging from a few hours to a few days (50), neutrophils are perpetually synthesized in the bone marrow and then dispatched into circulation. However, it's crucial to mention that neutrophils do not solely depend on this circulation for their immunological function. Current research underscores the significant role of marginated and tissue-resident neutrophils as first responders during infections and tissue injuries (51–53). These neutrophils swiftly counteract the spread of pathogens, thereby organizing the subsequent immune response. Once activated by chemotactic factors such as cytokines and chemokines, neutrophils navigate their way to the infection or inflammation site to eliminate pathogens employing a multitude of mechanisms (54). Their life cycle concludes with programmed cell death, or apoptosis, post which phagocytic cells, for instance, macrophages, clear them (55). This clearance of apoptotic neutrophils is paramount for inflammation resolution and prevention of tissue damage (56). Recent evidence indicates that aging significantly impacts neutrophil functionality, potentially triggering immune dysregulation. Aging is seemingly linked to a decline in neutrophil efficiency, as observed in the impairment of phagocytosis and oxidative burst, diminished cytokine production, and altered chemotaxis (57). Such alterations heighten the susceptibility to infections and inflammatory diseases in the elderly (58–60). Additionally, aging can reconfigure the gene expression profile of neutrophils, impacting their response to stimuli. Several factors, including changes in the bone marrow microenvironment, fluctuating levels of circulating hormones and cytokines, and cellular damage accumulation, are hypothesized to drive these age-related alterations in neutrophil function (61, 62). Therefore, it is crucial to consider the effect of aging on neutrophil functionality while conceptualizing new therapeutic interventions for infections and inflammatory conditions (63). Further investigations are warranted to unravel the mechanisms propelling age-induced changes in neutrophil function, thereby enabling the development of strategies to modulate neutrophil functionality in older adults. **Table 2** compares the impact of aging on various neutrophil functions, including phagocytosis, cytokine production, and ROS production, emphasizing the importance of considering the impact of aging on immune function.

**Table 2:** summarizing the evidence for different neutrophil-targeted therapies.

Therapy	Mechanism of Action	Preclinical Results	Clinical Trial Results	Potential Side Effects	Reference
Anti-IL-8 antibodies	Block IL-8-mediated neutrophil recruitment	Reduced lung injury in animal models of ARDS	Ongoing clinical trials	Potential immunosuppression	(64–67)
Dornase alfa	Degrade NETs and reduce inflammation	Improved lung function in COVID-19 patients	Improved lung function in small clinical trial	Potential respiratory tract infection	(68–72)
CXCR2 antagonists	Block CXCR2-mediated neutrophil recruitment	Reduced inflammation and improved survival in animal models of sepsis	Phase 2 clinical trial in patients with COPD ongoing	Potential impaired wound healing	(73–77)
MPO inhibitors	Block MPO-mediated neutrophil activation	Reduced tissue damage in animal models of stroke and myocardial infarction	Phase 1 clinical trial ongoing	Potential immunosuppression	(78–82)
Neutrophil elastase inhibitors	Block neutrophil elastase-mediated tissue damage	Reduced lung injury in animal models of ARDS	Phase 2 clinical trial in patients with COVID-19 ongoing	Potential impaired wound healing	(83–86)

## 3 Neutrophil heterogeneity

Discuss the concept of neutrophil heterogeneity and its implications

Recent studies have revealed that neutrophils are a heterogeneous population of cells with distinct subtypes that differ in their morphology, gene expression profile, and functional properties (87). The concept of neutrophil heterogeneity has important implications for our understanding of the immune response and the pathogenesis of various diseases (88). For example, different subtypes of neutrophils may have different functions in the immune response, with some subtypes being more effective at phagocytosis and others being more efficient at producing reactive oxygen species (89). Moreover, neutrophil heterogeneity may contribute to the development of chronic inflammation and autoimmunity, as some subtypes of neutrophils have been shown to be involved in the pathogenesis of these conditions (90). The identification of neutrophil subtypes has been facilitated by advances in single-cell genomics and proteomics techniques, which allow for the characterization of individual cells at the molecular level (91). The discovery of neutrophil heterogeneity has opened up new avenues for research into the immune response and the development of new therapies for inflammatory and autoimmune diseases (92). The potential of neutrophil-targeted therapies in various diseases is summarized in **Table 3**, which lists the mechanism of action, preclinical and clinical trial results, and potential side effects of different therapies. Recent advances in single-cell genomics and proteomics techniques have enabled the identification and characterization of different subtypes of neutrophils (106). These subtypes are distinguished by differences in their gene expression profiles, cell surface markers, and functional properties (107). It is notable that low-density neutrophils (LDNs) and high-density neutrophils (HDNs) were first identified back in 1986 (108). More recent studies have expanded our understanding of these subtypes, revealing that LDNs are typically more abundant in patients with autoimmune and inflammatory diseases and are associated with increased production of pro-inflammatory cytokines (109). In contrast, HDNs are more efficient at phagocytosis and have a higher oxidative burst capacity (110). Other subtypes of neutrophils have also been identified based on differences in their response to cytokines and chemokines (111). For example, neutrophils primed with interferon-gamma (IFN-γ) have been shown to be more efficient at phagocytosis and killing of bacteria (112). The functional differences between these subtypes of neutrophils have important implications for our understanding of the immune response and the development of new therapies for inflammatory and autoimmune diseases (113). Recent advances in the field of neutrophil biology have clearly underlined the heterogeneous nature of these critical immune cells (52). Neutrophils are no longer considered a homogenous population, but rather a highly versatile group with subsets that differ in their phenotypes and functions (114), crucial in maintaining homeostasis as well as responding to disease states. The heterogeneity of neutrophils can be influenced by several factors including the nature of the stimulus, the microenvironment, and their maturity (115). For example, research has highlighted the existence of low-density neutrophils (LDNs) and high-density neutrophils (HDNs) (116), which can be distinguished based on their buoyant density, morphological, and functional characteristics (117). LDNs are generally associated with chronic inflammation and are known to possess pro-inflammatory characteristics (117), while HDNs are usually found in healthy individuals, carrying out regular neutrophil functions such as phagocytosis and degranulation (46). Furthermore, the process of aging also significantly impacts neutrophil heterogeneity, introducing another level of complexity (118). The aforementioned heterogeneity plays a critical role during wound healing; different subsets of neutrophils have been found to be involved at various stages of wound healing (119), from the early inflammatory phase, where they act as the first line of defense against potential pathogens, to later stages, where they aid in tissue repair and regeneration (57, 120, 121). Therefore, a comprehensive understanding of neutrophil heterogeneity, along with the factors influencing it, is crucial for the elucidation of the complex role these cells play in health and disease. Recent studies have suggested that aging can impact neutrophil heterogeneity and contribute to immune dysregulation (120). For example, aging has been associated with changes in the gene expression profile of neutrophils, which may alter their functional properties and contribute to immune dysfunction (121). Moreover, aging can lead to the accumulation of cellular damage, which may affect the ability of neutrophils to respond to stimuli (57). These changes may also affect the balance between different subtypes of neutrophils, leading to an altered immune response (122). For example, aging has been associated with a shift towards pro-inflammatory neutrophil subtypes, which may contribute to chronic inflammation and increased susceptibility to infections (123). Additionally, aging can affect the bone marrow microenvironment, which may impact the production and differentiation of neutrophils (124). These findings highlight the importance of considering the impact of aging on neutrophil heterogeneity when developing new therapies for infectious and inflammatory diseases in older adults (125). Further research is needed to better understand the mechanisms underlying the impact of aging on neutrophil heterogeneity and immune function. Recent progress in research has highlighted the potential effects of aging and neutrophil heterogeneity on COVID-19, offering valuable insights into disease pathogenesis and clinical outcomes. Aging is associated with immunosenescence, a gradual decline in immune system function, which can impair the body's ability to effectively combat viral infections such as COVID-19. This age-related decline in immune response is of particular concern as it may contribute to increased disease severity and poorer outcomes in older individuals. Additionally, studies have revealed that neutrophils, a type of white blood cell crucial for immune defense, exhibit significant heterogeneity in their response to viral infections. This heterogeneity can impact the immune response to COVID-19, leading to variations in disease progression and patient outcomes. Understanding the underlying mechanisms and functional differences in neutrophil subsets holds promise for developing targeted therapeutic interventions and personalized treatment strategies for COVID-19 patients. Aging is a significant risk factor for severe outcomes in many infectious diseases, including COVID-19. The major contributor to this increased risk is immunosenescence, a state of gradual immune system deterioration that arises naturally with age. Immunosenescence impacts both the innate and adaptive arms of the immune response, including neutrophil function.

**Table 3:** Comparing the impact of aging on neutrophil function.

Function	Impact of Aging	Mechanisms	Implications	Reference
Phagocytosis	Reduced efficiency	Decreased expression of phagocytic receptors and ROS production	Increased susceptibility to infections	(93, 94)
Chemotaxis	Impaired	Decreased expression of chemotactic receptors	Delayed wound healing and impaired immune response	(95, 96)
Cytokine production	Altered	Increased pro-inflammatory cytokine production, decreased anti-inflammatory cytokine production	Increased chronic inflammation and impaired immune response	(97–99)
NET formation	Increased	Increased NET formation and decreased clearance	Increased thrombosis and tissue damage	(100–102)
ROS production	Altered	Decreased production, increased susceptibility to oxidative stress	Increased tissue damage and impaired immune response	(103–105)

## 4 Neutrophils and COVID-19

### 4.1 Summarize the current understanding of neutrophil involvement in COVID-19 pathogenesis

COVID-19 is a respiratory illness caused by the SARS-CoV-2 virus that has rapidly spread across the globe (126), leading to a global pandemic. Recent studies have suggested that neutrophils may play a crucial role in the pathogenesis of COVID-19 (127). Neutrophils are rapidly recruited to the lungs of COVID-19 patients and have been shown to release large amounts of reactive oxygen species (ROS) and pro-inflammatory cytokines, which contribute to the development of acute respiratory distress syndrome (ARDS) and multi-organ failure (128). Moreover, neutrophil extracellular traps (NETs) have been identified in the lungs of COVID-19 patients, which can contribute to thrombosis and tissue damage (129). The dysregulated neutrophil response in COVID-19 may be driven by a combination of factors, including virus-induced immune dysregulation, cytokine storm, and bacterial co-infections (130). These findings suggest that targeting neutrophil activation and recruitment may be a promising strategy for the treatment of COVID-19 (131). Several ongoing clinical trials are currently investigating the efficacy of neutrophil-targeted therapies in COVID-19 treatment. As we delve deeper into the interplay of aging, neutrophil heterogeneity, and COVID-19 severity, we can observe distinct patterns. These patterns, summarized in **Table 1**, clearly show that the immunosenescence and the nature of the neutrophil response vary significantly across age groups. These variations can influence the severity of COVID-19 symptoms, which generally tend to be milder in younger adults and more severe in older individuals. This highlights the importance of age and immune function, specifically neutrophil behavior, in determining the clinical outcomes of COVID-19 (**Supplementary Table 1**). Neutrophils are the most abundant type of white blood cell and form an essential first line of defense against infections (132, 133). They are traditionally considered short-lived, reactive cells that rapidly respond to infection signals (134). However, recent studies have begun to reveal the complex and varied nature of neutrophil biology (132). Far from being a homogenous population, neutrophils can be differentiated into various subpopulations based on their phenotype, function, and the context of the immune response (46, 135). In the context of COVID-19, neutrophil heterogeneity appears to play a critical role (136). The study underlines the crucial role of neutrophils in COVID-19, identifying a link between increased immature neutrophil populations and disease severity, while suggesting potential therapies targeting neutrophil-induced tissue damage (136). Different neutrophil subsets can be found in patients with varying severity of the disease (137, 138). Some subsets are associated with a heightened inflammatory response, often linked to severe disease and negative outcomes (24, 139, 140), while other subsets appear to be more regulatory, promoting resolution of inflammation and tissue repair (19). These observations have led to the emerging concept of "neutrophil plasticity" – the ability of neutrophils to dynamically adapt their functions in response to changes in the environment (124, 141). The age-related decline in immune function can exacerbate the dysregulation of the neutrophil response, leading to an overactive, damaging immune reaction – a situation often seen in severe COVID-19 cases (8). This study probes the association between severe COVID-19 and the aging immune system, particularly focusing on the exacerbated dysregulation of neutrophil response in the elderly. It posits that interventions targeting age-associated pathways could fortify immunity across diverse age cohorts, hence potentially reducing fatalities and enduring disabilities triggered by the pandemic (8). The characteristic 'cytokine storm' in severe COVID-19, driven by a hyperactive immune response, can cause extensive tissue damage and organ failure, leading to critical illness or death (142, 143). Older individuals, due to their compromised immune system, are more susceptible to this dysregulated immune response, explaining, at least in part, their increased vulnerability to severe disease (144, 145). Understanding the relationship between aging, neutrophil heterogeneity, and COVID-19 progression is essential for developing targeted therapies (146). By identifying key mechanisms driving neutrophil behavior in the context of age and COVID-19, researchers may develop strategies to modulate the immune response, mitigating the harmful effects while promoting protective immunity (147). Such an approach could involve the use of pharmaceutical agents to modify neutrophil function or the design of personalized treatment strategies based on a patient's specific neutrophil subset profile (148, 149). In conclusion, the complexities of the aging immune system and the multifaceted nature of neutrophil biology hold both challenges and opportunities for tackling COVID-19. Further research in these areas has the potential to yield significant improvements in the clinical management of the disease, particularly for vulnerable older populations.

### 4.2 Discuss the potential mechanisms underlying neutrophil activation and recruitment in COVID-19

The mechanisms underlying neutrophil activation and recruitment in COVID-19 are complex and multifactorial (150). The SARS-CoV-2 virus is known to directly infect and activate immune cells, including neutrophils, through binding to the angiotensin-converting enzyme 2 (ACE2) receptor (151). This activation can lead to the release of cytokines and chemokines, which further recruit and activate neutrophils to the site of infection (152). Moreover, COVID-19 is associated with a cytokine storm, which is characterized by the overproduction of pro-inflammatory cytokines, such as interleukin-6 (IL-6) and tumor necrosis factor-alpha (TNF-α) (153). These cytokines can activate neutrophils and promote their recruitment to the lungs, where they contribute to the development of ARDS and tissue damage (154). Additionally, bacterial co-infections are common in COVID-19 patients, and the presence of bacterial products, such as lipopolysaccharides (LPS), can further activate and recruit neutrophils (154). The dysregulated neutrophil response in COVID-19 may be a result of a combination of these factors, and understanding the underlying mechanisms may lead to the development of new therapeutic approaches to target neutrophil activation and recruitment in COVID-19 (155).

### 4.3 Evaluate the evidence for neutrophil-targeted therapies in COVID-19 treatment

There is growing interest in the potential of neutrophil-targeted therapies as a strategy for the treatment of COVID-19 (156). Several preclinical studies have suggested that blocking neutrophil recruitment and activation may improve clinical outcomes in COVID-19. For example, the use of anti-IL-8 antibodies (10), which block the recruitment of neutrophils, has been shown to reduce lung injury in animal models of COVID-19 (157). Other approaches, such as the use of NET inhibitors, have also shown promise in preclinical studies (158). Moreover, several ongoing clinical trials are investigating the efficacy of neutrophil-targeted therapies in COVID-19 treatment (159). One example is the use of dornase alfa, an FDA-approved medication used in the treatment of cystic fibrosis, which has been shown to degrade NETs and improve lung function in COVID-19 patients (160). However, there are also concerns that targeting neutrophils may impair the immune response to the virus, and the long-term effects of these therapies are not yet fully understood. Therefore, further research is needed to evaluate the safety and efficacy of neutrophil-targeted therapies in COVID-19 treatment (159).

## 5 Neutrophils and wound healing

Neutrophils play a critical role in the early stages of the wound healing process (161). Once a tissue injury occurs, neutrophils are rapidly recruited to the site of the wound, where they remove debris and bacteria through phagocytosis and the release of reactive oxygen species (ROS) (162). Neutrophils also release cytokines and chemokines, which recruit other immune cells to the site of the wound and promote angiogenesis and tissue remodeling (163). However, the excessive accumulation of neutrophils can also contribute to tissue damage and impair the wound healing process (164). Therefore, the balance between the pro-inflammatory and anti-inflammatory functions of neutrophils is critical for optimal wound healing (165). Recent studies have also suggested that different subtypes of neutrophils may have distinct functions in the wound healing process (164), and that the dysregulation of neutrophil heterogeneity may contribute to impaired wound healing in some conditions, such as diabetes (18). These findings suggest that targeting neutrophil function and heterogeneity may have therapeutic potential for improving wound healing outcomes. Recent studies have suggested that aging and neutrophil heterogeneity can impact the wound healing process (46). Aging is associated with a decline in neutrophil function, including impaired phagocytosis and oxidative burst, reduced cytokine production, and altered chemotaxis (166). These changes may impair the ability of neutrophils to effectively remove debris and bacteria from the site of the wound, leading to delayed wound healing and increased susceptibility to infections (167). Moreover, aging can also alter the gene expression profile of neutrophils and affect their response to stimuli, which may contribute to impaired wound healing (168). In addition, recent studies have identified different subtypes of neutrophils that have distinct functions in the wound healing process, such as those that are more efficient at phagocytosis or cytokine production (169). The dysregulation of neutrophil heterogeneity may contribute to impaired wound healing in some conditions, such as diabetes, where an imbalance between different neutrophil subtypes has been reported (170). These findings highlight the importance of considering the impact of aging and neutrophil heterogeneity on the wound healing process when developing new therapies to improve wound healing outcomes (171). Further research is needed to better understand the mechanisms underlying these effects and develop strategies to modulate neutrophil function and heterogeneity in older adults and those with impaired wound healing (172). The development of neutrophil-targeted therapies may hold promise for improving wound healing outcomes (12), particularly in conditions where impaired neutrophil function or dysregulated neutrophil activation is implicated in delayed healing (173). Recent studies have investigated various approaches for targeting neutrophils in the context of wound healing, including the use of anti-inflammatory agents (174), such as corticosteroids, and the inhibition of neutrophil-derived ROS and proteases (175). For example, the use of the ROS scavenger, N-acetylcysteine (NAC), has been shown to promote wound healing in diabetic mice by reducing oxidative stress and promoting angiogenesis (176). In addition, the use of protease inhibitors, such as serpinB1, has been shown to improve wound healing outcomes by reducing the activity of neutrophil-derived proteases (177), which can impair tissue regeneration. Moreover, recent studies have also suggested that modulating the balance between different subtypes of neutrophils may have therapeutic potential for improving wound healing outcomes (178). However, the safety and efficacy of these therapies in humans is not yet fully understood, and further research is needed to optimize these approaches and evaluate their potential clinical utility (179).

### 5.1 Impacts of COVID-19 on wound healing: nutritional status, skin manifestations, and immunosuppression

In the wake of recent advancements, a better understanding of how severe COVID-19 illness can detrimentally affect a patient's nutritional status has been developed (180–182). Nutrition is an integral component of wound healing, providing the necessary elements for tissue repair and immune function (183–185). Patients suffering from severe COVID-19, however, may experience drastic changes in their nutritional status due to various factors such as decreased appetite (186), increased metabolic demand due to the infection, or digestive complications associated with the disease (187, 188). This state of malnutrition may subsequently impede the wound healing process (189). Insufficient intake of protein, for example, can hinder tissue synthesis, while deficiencies in vitamins and minerals can disrupt collagen formation and immune response (190, 191), both crucial for wound recovery. Hence, malnutrition not only delays wound healing but also escalates the risk of complications, such as infection or wound dehiscence (192, 193). This understanding underscores the need for thorough nutritional assessment and appropriate dietary interventions in managing wound care for COVID-19 patient (194, 195). In light of recent studies, it has been observed that some patients with severe COVID-19 exhibit skin manifestations (196, 197), such as rashes or pseudo-chilblain lesions, colloquially known as "COVID toes." These dermatological symptoms likely arise from the virus's interaction with cells in the skin or as part of the body's immune response to the virus (198, 199). However, the precise correlation between these skin changes and wound healing remains elusive (200, 201). Some theories suggest that the increased inflammatory response associated with these skin conditions could potentially affect the phases of wound healing, which are inflammation, proliferation, and remodeling (202, 203). For instance, an exaggerated inflammatory response might lead to prolonged or chronic inflammation, delaying the progression to the subsequent phases of wound healing (204–206). Furthermore, if COVID-19 affects the blood vessels in the skin, as has been suggested by the presentation of pseudo-chilblain lesions (206, 207), this could impair the delivery of oxygen and nutrients essential for wound healing (208, 209). Ongoing research aims to elucidate the mechanisms underlying these observations, which will be crucial in tailoring wound care strategies for patients with severe COVID-19. Progressing research has begun to understand the intersection of COVID-19 and the immune system's responses, particularly regarding wound healing (210). Current research explores how COVID-19, immune responses, and wound healing are interlinked. Chronic conditions like MetS and T2DM often cause inflammation (211). COVID-19, due to an atypical immune response, triggers a unique cytokine storm, exacerbating inflammation. The prolonged inflammatory responses by SARS-CoV-2 can result in chronic inflammation and potential damage, such as fibrosis and pancreatic islet apoptosis (212). One of the significant discoveries is that COVID-19 infection often results in a reduced number of lymphocytes - a type of white blood cell that is vital in the immune response (213, 214). This is critical because lymphocytes, including T-cells and B-cells, play a pivotal role in the wound healing process, which includes phases of inflammation, proliferation, and remodeling (215, 216). They help orchestrate other cells' activities, release cytokines, and aid in combating potential infections at the wound site (184, 217). Therefore, a reduction in lymphocyte count, or immunosuppression, due to COVID-19 can potentially delay or impair the wound healing process (218, 219). This has significant implications for patient care, particularly for those who may require surgery or those with pre-existing wounds. Further research is needed to fully understand this process and develop strategies to support wound healing in the context of COVID-19 (220, 221). Considering recent progress in understanding the effects of severe COVID-19 on wound healing, a multi-faceted approach is necessary for future therapy development and improvement of mechanisms. Enhanced nutritional support should be a focus area, considering the role of malnutrition in impeding wound healing (222). Novel strategies to manage appetite loss and digestion complications should be explored along with high-protein diets or supplements and adequate micronutrient intake. Simultaneously, dermatological treatments should be considered for patients exhibiting skin manifestations (199, 223, 224). Unraveling the links between skin changes and wound healing could lead to targeted topical treatments that manage inflammation and support the skin's natural healing process. Furthermore, immunotherapy might be crucial given COVID-19's impact on lymphocyte counts. Therapies to restore lymphocyte function or count, such as immunomodulatory drugs or cytokine therapies, may offer new avenues for supporting wound healing (225). Personalized medicine, combining nutritional support, skin care, and immunotherapy, could maximize patient outcomes, necessitating further research into individual variations. Comprehensive and ongoing research into these mechanisms will be pivotal to develop robust therapeutic strategies and improve patient recovery and quality of life in the context of COVID-19.

## 6 Conclusion

In this review, we discussed recent progress in understanding the role of neutrophils in various physiological and pathological processes, including immune response, aging, COVID-19, and wound healing. We highlighted the importance of considering the heterogeneity of neutrophils and its impact on immune function and disease pathogenesis. We also reviewed the potential of neutrophil-targeted therapies in various diseases, including COVID-19, and discussed the potential effects of aging and neutrophil heterogeneity on wound healing outcomes. The research in this field has identified novel therapeutic targets and provided insights into the underlying mechanisms of disease pathogenesis. The implications of this research for future studies and clinical practice are significant, as it may lead to the development of more targeted and effective therapies for infectious and inflammatory diseases and improved wound healing outcomes. Moreover, the identification of neutrophil subtypes and the characterization of their functions may facilitate the development of personalized medicine approaches. However, further research is needed to fully understand the complexity of neutrophil biology and its impact on various disease processes.

## Author contributions

WW: writing, editing, reviewing, and conceptualization. CX: writing, editing, reviewing, and conceptualization. LY: writing, editing, reviewing, and conceptualization. CL: reviewing and editing. CC: reviewing, editing, and supervision. ZQ: writing and revision of the manuscript. YL: writing and conceptualization. YZ: revision and managing the project. All authors contributed to the article and approved the submitted version.
