# Supplementary material for: Neutrophil heterogeneity and aging: implications for COVID-19 and wound healing
Source: Front Immunol. 2023 Nov 28;14:1201651. doi: 10.3389/fimmu.2023.1201651 (PMC10715311; doi:10.3389/fimmu.2023.1201651)
Supplement: Supplementary file 1 [file Table_1.docx]

| Age Group | Immunosenescence | Neutrophil Response | COVID-19 Severity | Reference |
| --- | --- | --- | --- | --- |
| Young Adults | Low levels of immunosenescence, robust immune response | Predominantly regulatory neutrophil subsets, efficient resolution of inflammation | Generally mild to moderate symptoms | [1, 2] |
| Middle-aged Adults | Moderate levels of immunosenescence, somewhat compromised immune response | Mixed neutrophil subsets, some pro-inflammatory | Moderate to severe symptoms in some cases | [3-9] |
| Older Adults | High levels of immunosenescence, compromised immune response | Predominantly pro-inflammatory neutrophil subsets, heightened inflammatory response | Often severe symptoms, increased risk of critical illness | [231-233] |

Supplementary Table 1: Aging, Neutrophil Heterogeneity, and COVID-19 Severity

[1] L.L. Cunha, S.F. Perazzio, J. Azzi, P. Cravedi, L.V. Riella, Remodeling of the Immune Response With Aging: Immunosenescence and Its Potential Impact on COVID-19 Immune Response, Frontiers in immunology 11 (2020) 1748.

[2] A. Santoro, E. Bientinesi, D. Monti, Immunosenescence and inflammaging in the aging process: age-related diseases or longevity?, Ageing research reviews 71 (2021) 101422.

[3] A. Abdoli, H.M. Ardakani, Helminth infections and immunosenescence: The friend of my enemy, Experimental gerontology 133 (2020) 110852.

[4] M. Bonifazi, F. Mei, E. Skrami, L.L. Latini, D. Amico, E. Balestro, F. Bini, F. Bonifazi, A. Caminati, P. Candoli, S. Cinti, S. Contucci, A. Di Marco Berardino, S. Harari, G. Levi, S. Lococo, V. Menditto, G. Marchetti, S. Piciucchi, V. Poletti, C. Ravaglia, M. Saetta, G. Svegliati-Baroni, S. Tomassetti, M. Tamburrini, A. Zanforlin, U. Zuccon, L. Zuccatosta, S. Gasparini, F. Carle, Predictors of Worse Prognosis in Young and Middle-Aged Adults Hospitalized with COVID-19 Pneumonia: A Multi-Center Italian Study (COVID-UNDER50), Journal of clinical medicine 10(6) (2021).

[5] A.L. Mueller, M.S. McNamara, D.A. Sinclair, Why does COVID-19 disproportionately affect older people?, Aging 12(10) (2020) 9959-9981.

[6] S. Niu, S. Tian, J. Lou, X. Kang, L. Zhang, H. Lian, J. Zhang, Clinical characteristics of older patients infected with COVID-19: A descriptive study, Archives of gerontology and geriatrics 89 (2020) 104058.

[7] J.E. McElhaney, C.P. Verschoor, M.K. Andrew, L. Haynes, G.A. Kuchel, G. Pawelec, The immune response to influenza in older humans: beyond immune senescence, Immunity & ageing : I & A 17 (2020) 10.

[8] J. Bischof, F. Gärtner, K. Zeiser, R. Kunz, C. Schreiner, E. Hoffer, T. Burster, U. Knippschild, M. Zimecki, Immune Cells and Immunosenescence, Folia biologica 65(2) (2019) 53-63.

[9] Z. Liu, Q. Liang, Y. Ren, C. Guo, X. Ge, L. Wang, Q. Cheng, P. Luo, Y. Zhang, X. Han, Immunosenescence: molecular mechanisms and diseases, Signal transduction and targeted therapy 8(1) (2023) 200.
